# Mef2c Exacerbates Neuron Necroptosis via Modulating Alternative Splicing of Cflar in Ischemic Stroke With Hyperlipidemia

**DOI:** 10.1111/cns.70144

**Published:** 2024-12-08

**Authors:** Ruqi Li, Tianchen Huang, Jianpo Zhou, Xiansheng Liu, Gan Li, Yueman Zhang, Yunlu Guo, Fengshi Li, Yan Li, Arthur Liesz, Peiying Li, Zhenghong Wang, Jieqing Wan

**Affiliations:** ^1^ Cerebrovascular Diseases Center, Department of Neurosurgery Renji Hospital, Shanghai Jiao Tong University School of Medicine Shanghai China; ^2^ Department of Anesthesiology, Key Laboratory of the Ministry of Education Renji Hospital, Shanghai Jiao Tong University School of Medicine Shanghai China; ^3^ Clinical Research Center Renji Hospital, Shanghai Jiao Tong University School of Medicine Shanghai China; ^4^ Institute for Stroke and Dementia Research (ISD), University Hospital, LMU Munich Germany; ^5^ Munich Cluster for Systems Neurology (SyNergy) Munich Germany

**Keywords:** alternative splicing, Cflar, hyperlipidemia, Mef2c, necroptosis, stroke

## Abstract

**Aim:**

Hyperlipidemia is a common comorbidity of stroke patients, elucidating the mechanism that underlies the exacerbated ischemic brain injury after stroke with hyperlipidemia is emerging as a significant clinical problem due to the growing proportion of hyperlipidemic stroke patients.

**Methods:**

Mice were fed a high‐fat diet for 12 weeks to induce hyperlipidemia. Transient middle cerebral artery occlusion was induced as a mouse model of ischemic stroke. *Emx1* ^*Cre*^ mice were crossed with *Mef2c* ^*fl/fl*^ mice to specifically deplete Mef2c in neurons.

**Results:**

We reported that hyperlipidemia significantly aggravated neuronal necroptosis and exacerbated long‐term neurological deficits following ischemic stroke in mice. Mechanistically, Cflar, an upstream necroptotic regulator, was alternatively spliced into pro‐necroptotic isoform (Cflar_R_) in ischemic neurons of hyperlipidemic mice. Neuronal Mef2c was a transcription factor modulating Cflar splicing and upregulated by hyperlipidemia following stroke. Neuronal specific Mef2c depletion reduced cerebral level of Cflar_R_ and cFLIP_R_ (translated by Cflar_R_), while mitigated neuron necroptosis and neurological deficits following stroke in hyperlipidemic mice.

**Conclusions:**

Our study highlights the pathogenic role of Cflar_R_ splicing mediated by neuronal Mef2c, which aggravates neuron necroptosis following stroke with comorbid hyperlipidemia and proposes Cflar_R_ splicing as a potential therapeutic target for hyperlipidemic stroke patients.

## Graphical abstract

Hyperlipidemia is a common comorbidity of stroke, which exacerbated ischemic brain injury. We found that hyperlipidemia promoted alternative splicing of Cflar_R_ via upregulating Mef2c expression in ischemic neurons, thus aggravated neuron necroptosis and neurological deficits following ischemic stroke. This pathogenic splicing process is a promising target for hyperlipidemic stroke patients.

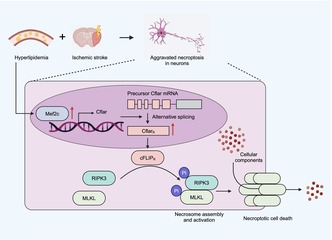

## 1 Introduction

Ischemic stroke affects over 70 million people worldwide [1], posing an enormous burden on patient families and the society due to disability, cognitive impairment, and other complications [2]. The severity of neurological deficits and long‐term outcome vary considerably among stroke patients with different comorbidities, such as hypertension, diabetes, and hyperlipidemia. Hyperlipidemia is a common comorbidity in stroke patients, affecting approximately 23%–53% of the patients [3, 4]. Moreover, hyperlipidemia is an independent risk factor of neurological deficits and disability in ischemic stroke patients [5, 6, 7, 8]. Previous studies demonstrated that neurological function of stroke patients with hyperlipidemia is worse in comparison to non‐hyperlipidemic stroke patients [7, 9]. Therefore, unveiling the mechanisms that underlie the clinically observed exacerbated brain injury after ischemic stroke in patients with hyperlipidemia is important for the development of novel treatment strategies for these patients.

Necroptosis is a cell death mechanism characterized by activation of receptor interacting protein kinase 1 (RIPK1), RIPK3, and mixed lineage kinase domain like protein (MLKL). Necroptosis is generally triggered by tumor necrosis factor receptor (TNFR) in response to inflammation [10, 11]. Necroptosis not only impairs physiological neuronal functions, but also exacerbates neuroinflammation and brain damage by promoting neuronal release of damage‐associated molecular patterns (DAMPs), involving high mobility group box 1 and genome DNA [12, 13]. It was previously demonstrated that ischemic stroke can induce neuronal necroptosis, leading to deterioration of neurological deficits [14, 15, 16].

The cellular FADD‐like interleukin‐1β‐converting enzyme inhibitory protein (cFLIP) is a strong endogenous regulator of necroptosis, and it is encoded by CASP8 and FADD‐like apoptosis regulator (*Cflar*). Cflar long isoform (Cflar_L_) is a group of most common *Cflar* transcripts in mice, while in certain conditions the precursor RNA can be alternatively spliced into Cflar Raji isoform (Cflar_R_) [17]. The corresponding protein isoforms, cFLIP_L_ and cFLIP_R_, N‐terminally contain two death effector domains which enable their binding to procaspase 8. This forms cFLIP‐procaspase 8 heterodimer [17]. The longer isoform, cFLIP_L_, C‐terminally contains extra caspase‐like domains, which activate procaspase 8, leading to RIPK1 inactivating and ripoptosome disassembling [18, 19]. In contrast, cFLIP_R_ effectively blocks procaspase 8 activation due to its lack of the caspase‐like domain, thus promotes necroptosis [17, 20]. Cflar splicing was reported to be mediated by transcription factors [21]. Moreover, a previous study demonstrated that the relative Cflar_R_ level in brain was correlate with the severity of ischemic brain injury in rodent, suggesting that Cflar splicing is a critical regulatory step of cerebral cell death [22]. Thus, it is important to reveal the role and the regulatory mechanism of Cflar splicing following ischemic stroke.

Here we found that hyperlipidemia induced by high‐fat diet (HFD) increases the expression of myocyte enhancer factor 2c (Mef2c), a transcription factor regulating cell fate and inflammatory response in ischemic neurons, and found that Mef2c promotes the alternative splicing of Cflar toward Cflar_R_, which consequently aggravated neuronal necroptosis. Neuron‐specific ablation of Mef2c reduced Cflar_R_ and cFLIP_R_ expression, thus alleviating neuronal necroptosis and neurological deficits in HFD‐treated mice after stroke. Our results reveal that Mef2c‐regulated alternative Cflar splicing would be a potent neuroprotective target for stroke patients with hyperlipidemia.

## 2 Methods

All animal experiments were approved by the Renji Hospital Institutional Animal Care and Use Committee, and performed under the Institutional Guide for the Care and Use of Laboratory Animals, which is accorded with Animal Research: Reporting in Vivo Experiments (ARRIVE) guidelines [23]. Detailed descriptions of the methods are available in the Supporting Information. The data that support the findings of this study are available from the corresponding author upon reasonable request.

## 3 Results

### 3.1 HFD Aggravated Neuronal Necroptosis and Neurological Deficits in MCAO Mice

**FIGURE 1:**
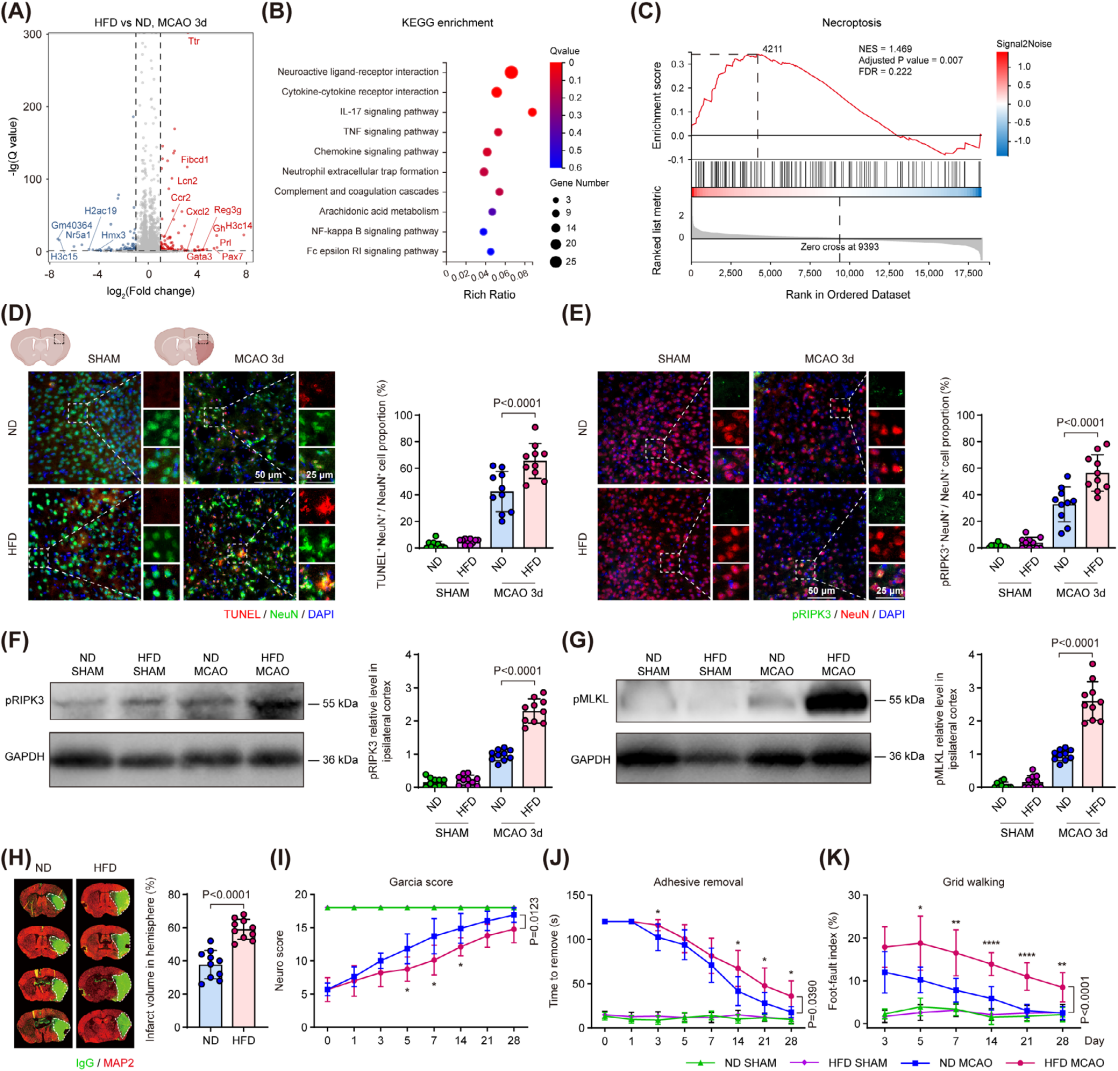
HFD aggravated neuronal necroptosis and exacerbated neurological impairments following MCAO. (A) Volcano plot illustrated cortical DEGs between HFD‐ and ND‐treated mice 3 days following MCAO. *n* = 3. (B) Bubble plot showed KEGG enrichment results using DEGs between HFD‐ and ND‐treated mice 3 days following MCAO. (C) GSEA illustrated that necroptosis pathway was activated in the cortex tissue of HFD‐treated mice 3 days following MCAO. (D, E) Representative image of neuron necroptosis in ND‐ and HFD‐treated mice 3 days after MCAO. Quantification of TUNEL^+^ NeuN^+^ (D) and phosphorylated RIPK3^+^ (pRIPK3^+^) NeuN^+^ (E) neuron percentage in the peri‐infarct region. *n* = 10, One‐way ANOVA with Tukey's multiple comparison test. (F, G) Representative immunoblots for kinases mediating necroptosis in ND‐ and HFD‐treated mice 3 days following MCAO. Quantitative analysis of pRIPK3 (F) and phosphorylated MLKL (pMLKL) (G) relative level in the ischemic cortex. *n* = 10, One‐way ANOVA with Tukey's multiple comparison test. (H) Representative images and quantification of IgG and MAP2 double staining in the brain of ND‐ and HFD‐treated mice 3 days following MCAO. *n* = 10, unpaired Student's *t‐*test. (I, K) Post‐stroke neurological assessment, including modified Garcia Score (I), adhesive removal test (J) and forelimb foot‐faults index (K), in ND‐ and HFD‐treated mice through 28 days following MCAO. *n* = 8–10, Two‐way ANOVA with *post hoc* Bonferroni test, *p < 0.05, **p < 0.01, ****p < 0.0001 HFD MCAO versus ND MCAO. All data are presented as means ± SD. ANOVA, analysis of variance; DEGs, Differentially expressing genes; GSEA, gene set enrichment analysis; HFD, high‐fat diet; IgG, immunoglobulin G; KEGG, Kyoto Encyclopedia of Genes and Genomes; ND, normal diet; MAP2, microtube‐associated protein 2; MCAO, middle cerebral artery occlusion; SD, standard deviation; TdT‐mediated dUTP nick‐end labeling (TUNEL).

**FIGURE 2:**
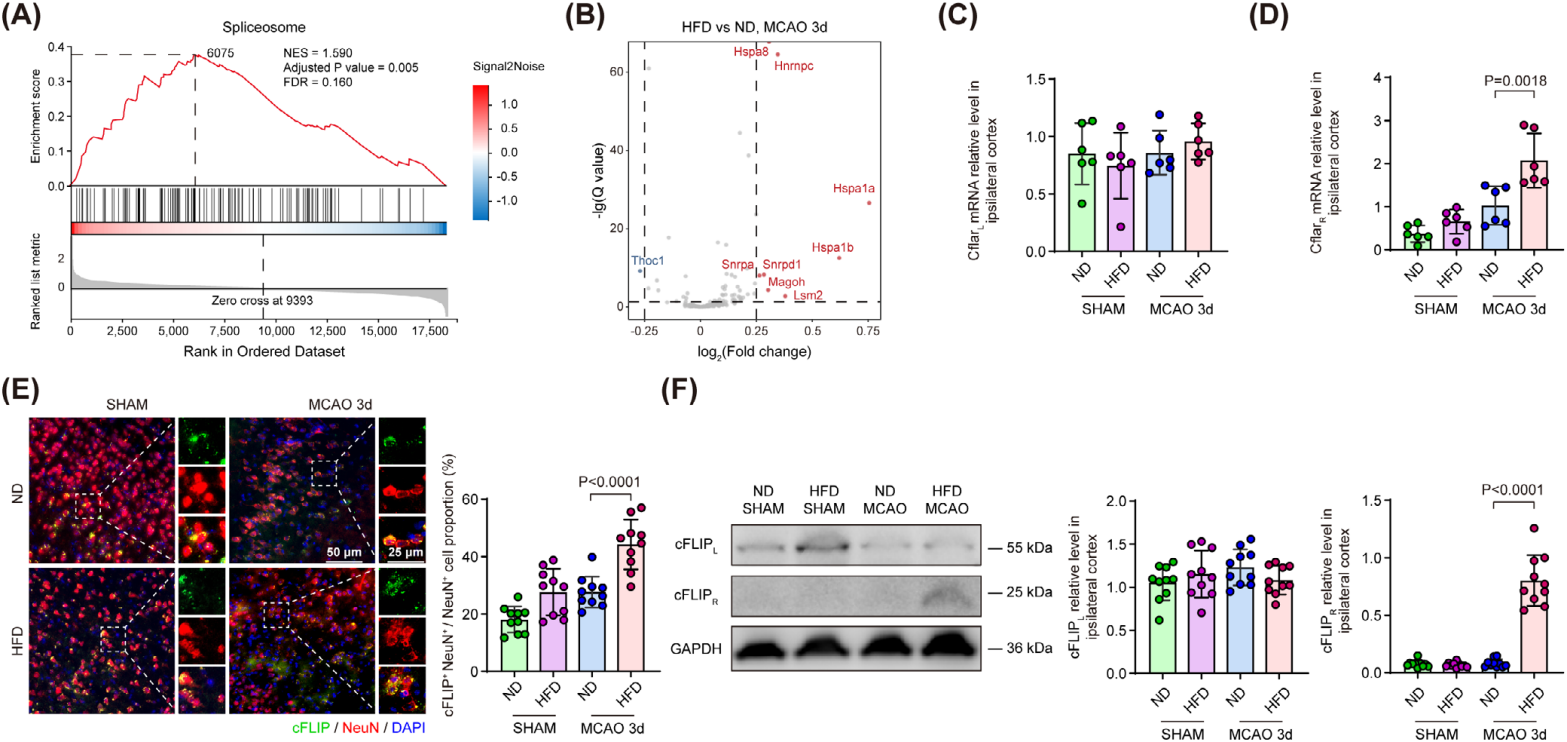
HFD upregulated Cflar_R_ splicing isoform, an upstream pro‐necroptotic regulator, in neurons following MCAO. (A) GSEA illustrated that spliceosome pathway was activated in the cortex tissue of HFD‐treated mice 3 days following MCAO. (B) Volcano plot illustrated top genes in spliceosome gene set between HFD‐ and ND‐treated mice 3 days following MCAO. (C, D) Relative mRNA expression of Cflar_L_ (B) and Cflar_R_ (C) examined by qPCR in the cortex of ND‐ and HFD‐treated mice 3 days following MCAO. *n* = 6, One‐way ANOVA with Tukey's multiple comparison test. (E) Representative images of cFLIP localized in neurons of ND‐ and HFD‐treated mice 3 days following MCAO. Quantification of cFLIP^+^ NeuN^+^ neuron percentage in the peri‐infarct region. *n* = 10, One‐way ANOVA with Tukey's multiple comparison test. (F) Representative immunoblot for cortical cFLIP_L_ and cFLIP_R_ level of ND‐ and HFD‐treated mice 3 days following MCAO. Quantitative analysis of cFLIP_L_ and cFLIP_R_ relative level in ischemic cortex. *n* = 10, One‐way ANOVA with Tukey's multiple comparison test. All data are presented as means ± SD.

To investigate transcriptome alternation associated with hyperlipidemia, we harvested cortex tissue from mice receiving normal diet (ND) and HFD for 12 weeks to conduct next‐generation sequencing 3 days following ischemic stroke induced by transient middle cerebral artery occlusion (MCAO). Differential expression analysis revealed that genes related to inflammatory activation (Ccr2 and Cxcl2) [24, 25], molecule transport (Ttr and Lcn2) [26, 27], and transcriptional control (H3c14, Gata3, and Pax7) [28, 29, 30] were significantly upregulated in HFD‐treated mice (Figure 1A). The subsequent KEGG enrichment analysis revealed a significant enrichment in pathways associated with neuroactive ligand–receptor interaction and several neuroinflammation‐associated pathways (Figure 1B). Further GSEA demonstrated that necroptosis, an inflammation‐responding and TNF‐triggering cell death progress, was significantly activated in the cortex of HFD‐treated mice 3 days following MCAO (Figure 2C). These results suggested that HFD aggravated neuroinflammation and enabled the excessive activation of necroptosis following ischemic stroke.

To examine the impact of HFD on neuronal cell death and necroptosis after ischemic stroke, we double labeled NeuN (neuronal specific marker) with TUNEL (labeling truncated DNA representing cell death) and phosphorylated RIPK3 (pRIPK3, an activated kinase promoting necroptosis), and found that both percentages of TUNEL^+^ NeuN^+^ and pRIPK3^+^ NeuN^+^ neuron were significantly elevated in the peri‐infarct region of HFD‐treated mice compared to that in ND‐treated mice 3 days after MCAO (Figure 1D,E). Using immunoblot analysis, we found that the level of pRIPK3 and phosphorylated MLKL (pMLKL), two critical kinases that compose the necrosome complex, were significantly increased in the ischemic cortex of HFD‐treated mice compared to that of ND‐treated mice 3 days after MCAO (Figure 1F,G). These results suggested that HFD exacerbated neuronal necroptosis following ischemic stroke.

To investigate whether HFD aggravated ischemic injury and stroke outcome, we stained brain sections with immunoglobulin G (IgG) and microtubule‐associated protein 2 (MAP2), and found that the infarct volume of HFD‐treated mice was significantly increased compared to that of ND‐treated mice 3 days following MCAO (Figure 1H). We also examined the post‐stroke neurological deficits in the ND‐ and HFD‐treated mice with three sets of behavioral tests, including the mGarcia score, adhesive removal test and grid walking test up to 28 days following MCAO. We found that the behavioral function score of HFD‐treated mice was significantly impaired in comparison to controls, as indicated by a reduced neurological score (Figure 1I) and prolonged duration to sticker removal up to 28 days following stroke (Figure 1J). In the grid walking test, the foot‐fault index, which was calculated as (right impaired forelimb faults—left intact forelimb faults)/total forelimb faults of bilateral forelimbs, was significantly increased in HFD‐treated mice compared to that in ND‐treated mice up to 28 days after MCAO (Figure 1K). These results suggested that HFD exacerbates cerebral ischemic injury and worsen post‐stroke neurological deficits following ischemic stroke.

### 3.2 HFD Induced Alternative Splicing of Cflar Toward the Cflar_R_ Isoform, an Upstream Necroptotic Promotor, in Ischemic Neurons

To investigate the underlying mechanism that HFD aggravated neuronal necroptosis following ischemic stroke, we reviewed the above GSEA results and found that the spliceosome pathway was simultaneously activated with necroptosis in HFD‐treated group 3 days following MCAO (Figure 2A). Using differential expression analysis to identify top genes in spliceosome gene set, we found that genes responsible for splicing site recognition and spliceosome recruitment (Hnrnpc, Snrpa, Snrpd1, and Lsm2) [31, 32, 33], nuclear export of mRNA (Magoh) [34] and maintaining normal functions of spliceosome (Hspa1a, Hspa1b, and Hspa8) [35, 36] were upregulated in HFD‐treated mice 3 days following MCAO (Figure 2B). These results suggested that these genes could mediate the activation of alternative splicing process in ischemic cortex from HFD‐treated mice. The initiation of necroptosis has been reported under the regulation of Cflar alternative splicing [19, 37]. Therefore, we implemented quantitative polymerase chain reaction (qPCR) to analyze the expression level of Cflar mRNA variants in ischemic cortex of mice. We found that Cflar_L_ exhibited no significant difference between ND‐ and HFD‐treated mice 3 days following ischemic stroke (Figure 2C), but Cflar_R_ was significantly upregulated in HFD‐treated mice compared to that in ND‐treated mice 3 days following MCAO (Figure 2D). These results suggested HFD induced alternative splicing of Cflar and promoted Cflar_R_ generation in the ischemic brain.

To examine the impact of HFD on the expression level of neuronal cFLIP, the protein translated from Cflar mRNA, we co‐stained NeuN and cFLIP in the brain sections of ND‐ and HFD‐treated mice 3 days following ischemic stroke. We found that the percentage of cFLIP^+^ NeuN^+^ neuron was significantly increased in the peri‐infarct region of HFD‐treated mice compared to that in ND‐treated mice 3 days after MCAO (Figure 2E). Using western blot, we found that the expression level of cFLIP_L_ did not show significant changes between groups, while cFLIP_R_ was significantly upregulated in the ischemic cortex of HFD‐treated mice compared to that of ND‐treated mice 3 days after MCAO (Figure 2F). These results suggested HFD elevated neuronal cFLIP_R_ level but not cFLIP_L_ following ischemic stroke, which corroborated that HFD induced the alternative splicing of Cflar from the protein level.

### 3.3 Neuronal Mef2c Was Critical in Modulating Alternative Splicing of Cflar and UpRegulated in HFD‐Treated Mice Following MCAO

**FIGURE 3:**
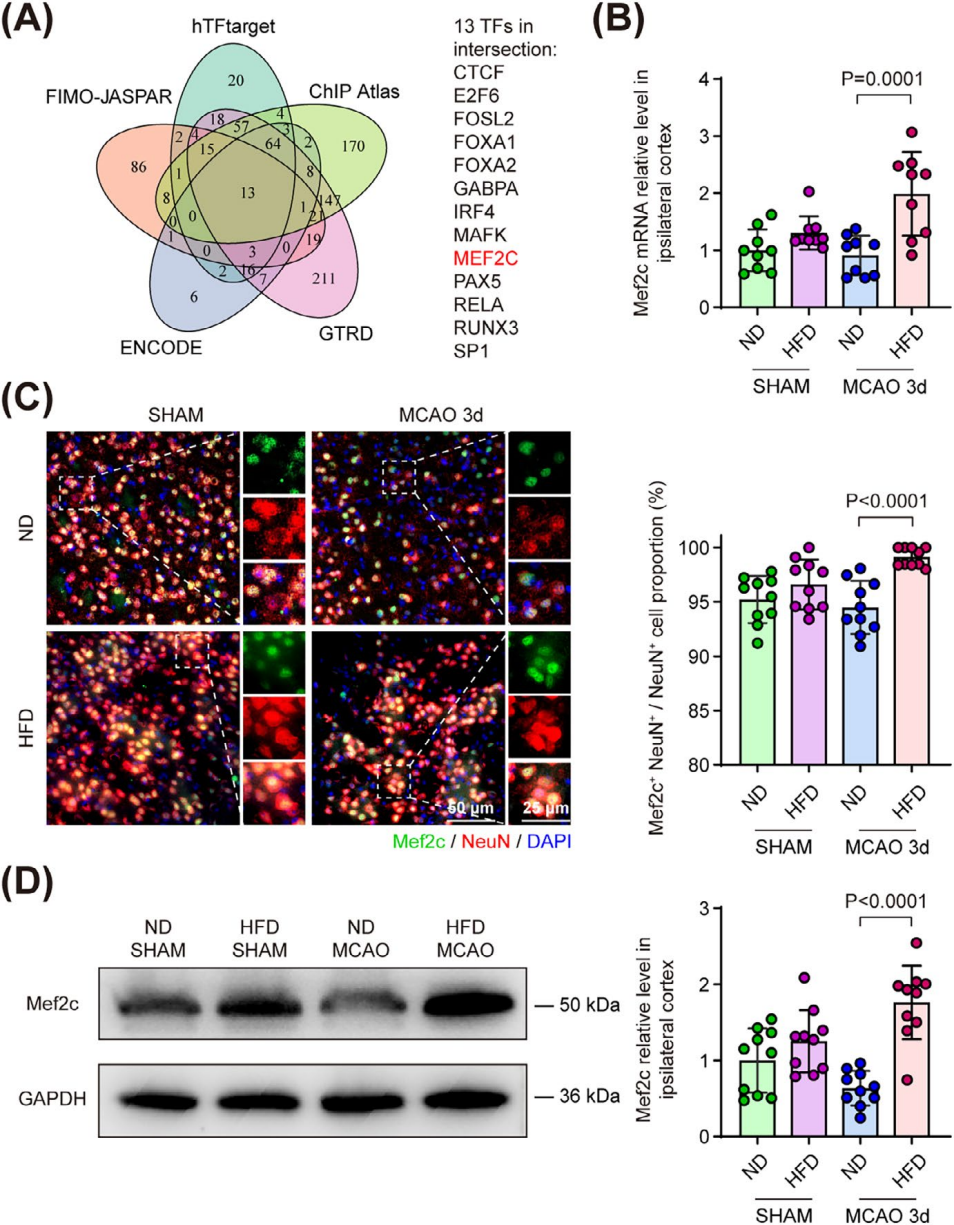
Neuronal Mef2c was upregulated in HFD‐treated stroke mice. (A) Venn plot illustrating the intersection of transcription factors (TFs) which were predicted to be involved in Cflar transcription using five public TF databases. (B) Relative mRNA expression of Mef2c examined by qPCR in the cortex of ND and HFD‐treated mice 3 days following MCAO. *n* = 9, One‐way ANOVA with Tukey's multiple comparison test. (C) Representative images of Mef2c localized in neurons of ND‐ and HFD‐treated mice 3 days following MCAO. Quantification of Mef2c^+^ NeuN^+^ neuron percentage in the penumbra area. *n* = 10, One‐way ANOVA with Tukey's multiple comparison test. (D) Representative immunoblot for cortical Mef2c level of ND‐ and HFD‐treated mice 3 days following MCAO. Quantitative analysis of Mef2c relative level in ischemic cortex. *n* = 10, One‐way ANOVA with Tukey's multiple comparison test. All data are presented as means ± SD.

The expression of Cflar splicing variants is modulated by diverse transcription factors (TF) varying from cell and tissue types [21]. Considering that *Cflar* gene exhibits considerate evolutionary conservation in sequence (homology between human and mouse *Cflar* gene sequences is 43.87%) and single‐nucleotide polymorphism related to splicing (e.g., rs10190751), we used a bioinformatic toolkit, which incorporated with well‐acknowledged human TF‐target online tools (including hTFtarget, ChIP Atlas, GTRD, ENCODE, and JASPAR) to match TF's motif sequence with the transcription starting site of *Cflar* and integrate retrieved results to predict TFs regulating *Cflar* expression [21, 38, 39]. Thirteen TFs were found in the intersection of results from five database (Figure 3A). Assessing the probability of these TFs binding to human *CFLAR* sequence with JASPAR database, we found that Mef2c had two specific binding sites in *CFLAR* with high confidence level (Table S1). Moreover, the expression of Mef2c was reported upregulated by long‐term treatment of HFD in myocardial cells [40]. Therefore, we assumed that Mef2c was most likely to regulate alternative splicing of Cflar following MCAO in the context of HFD.

Mef2c is a transcription factor recently identified as the key regulator of cell viability and neuroinflammation in several central nerve system diseases [41, 42, 43]. Using qPCR, we found that cortical Mef2c mRNA was upregulated in HFD‐treated mice compared to that in ND‐treated mice 3 days following MCAO (Figure 3B). To investigate the cell‐type expression pattern of Mef2c, we co‐stained Mef2c with several cerebral cell markers, including NeuN, Glial fibrillary acidic protein (GFAP, astrocyte), and ionized calcium‐binding adapter molecule 1 (Iba‐1, microglia) both in ND‐ and HFD‐treated mice brain sections. We found the percentage of Mef2c^+^ NeuN^+^/NeuN^+^ cells in peri‐infarct region exceeded 85% in both ND‐ and HFD‐treated groups under sham condition or 3 days following MCAO. The percentage of Mef2c^+^ neurons was significantly increased in HFD‐treated mice compared to that in ND‐treated mice 3 days following MCAO (Figure 3C). Nonetheless, no Mef2c^+^ signal was found in astrocytes of ND‐ and HFD‐treated mice 3 days following MCAO (Figure S1A). In accordance with previous reports studying Mef2c in microglia, we detected Mef2c signals in microglia [42, 44]. However, the percentage of Mef2c^+^Iba‐1^+^ microglia was not significantly changed in HFD‐treated mice compared to that in ND‐treated mice 3 days following MCAO (Figure S1B). Consistent with results of qPCR and immunofluorescence, we found that the cortical protein level of Mef2c was significantly increased in HFD‐treated mice compared to that in ND‐treated mice 3 days following MCAO (Figure 3D). These results suggested cerebral Mef2c was mainly expressed in neurons and upregulated by HFD.

### 3.4 Neuronal Specific Depletion of Mef2c ( *Emx1* ^*Cre*^ *Mef2c* ^*fl*^ ^*/fl*^) Suppressed HFD‐Induced Cflar_R_ Splicing Following MCAO

**FIGURE 4:**
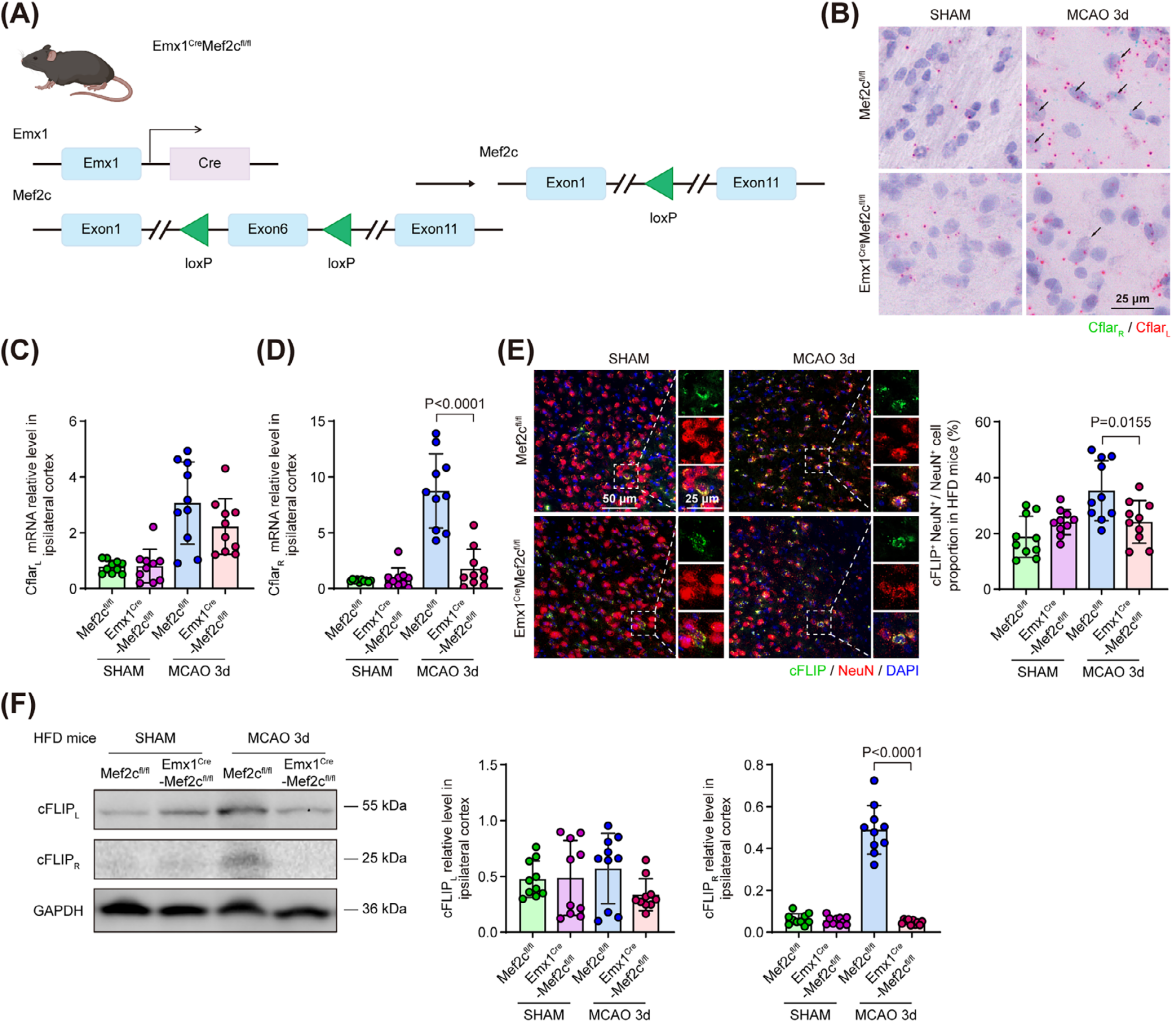
Neuronal depletion of Mef2c (*Emx1* ^*Cre*^ *Mef2c* ^*fl/fl*^) suppressed HFD‐induced Cflar_R_ splicing following MCAO. (A) Illustration of generating *Emx1* ^*Cre*^ *Mef2c* ^*fl/fl*^ conditional knockout mice. (B) Representative Basescope images of Cflar_L_ and Cflar_R_ mRNA localized in ischemic neurons of *Mef2c* ^*fl/fl*^ and *Emx1* ^*Cre*^ *Mef2c* ^*fl/fl*^ mice which were both treated with HFD and sacrificed 3 days after MCAO. Arrows pointing at Cflar_R_ signals. (C, D) Relative mRNA expression of Cflar_L_ (C) and Cflar_R_ (D) examined by qPCR in the cortex of *Mef2c* ^*fl/fl*^ and *Emx1* ^*Cre*^ *Mef2c* ^*fl/fl*^ mice (both HFD‐treated) 3 days following MCAO. *n* = 10, One‐way ANOVA with Tukey's multiple comparison test. (E) Representative images of cFLIP localized in neurons of *Mef2c* ^*fl/fl*^ and *Emx1* ^*Cre*^ *Mef2c* ^*fl/fl*^ mice (both HFD‐treated) 3 days following MCAO. Quantification of cFLIP^+^ NeuN^+^ neuron percentage in the peri‐infarct region. *n* = 10, One‐way ANOVA with Tukey's multiple comparison test. (F) Representative immunoblot for cortical cFLIP_L_ and cFLIP_R_ level of *Mef2c* ^*fl/fl*^ and *Emx1* ^*Cre*^ *Mef2c* ^*fl/fl*^ mice (both HFD‐treated) 3 days following MCAO. Quantitative analysis of cFLIP_L_ and cFLIP_R_ relative level in ischemic cortex. *n* = 10, One‐way ANOVA with Tukey's multiple comparison test. All data are presented as means ± SD.

To testify whether neuronal Mef2c was responsible for Cflar_R_ splicing in MCAO mice with HFD pre‐treatment, we used CRISPR‐Cas9 system to generate a *Mef2c* ^*fl/fl*^ transgenic line and crossed them with *Emx1* ^*Cre*^ mice to conditionally deplete Mef2c in neurons (Figure 4A). Neuronal Mef2c was effectively depleted in *Emx1* ^*Cre*^ *Mef2c* ^*fl/fl*^ mice 3 days following MCAO (Figure S2A,B). Using BaseScope to label Cflar_L_ and Cflar_R_ mRNA in situ, we detected Cflar_L_^+^ signals in peri‐infarct neurons in HFD pre‐treated *Mef2c* ^*fl/fl*^ and *Emx1* ^*Cre*^ *Mef2c* ^*fl/fl*^ mice undergoing sham or MCAO procedure. Meanwhile, we found stronger Cflar_R_^+^ signals emerged in HFD‐treated *Mef2c* ^*fl/fl*^ mice compared to HFD‐treated *Emx1* ^*Cre*^ *Mef2c* ^*fl/fl*^ mice 3 days following MCAO, but no Cflar_R_^+^ signal was observed in sham groups (Figure 4B). Using cortex tissue for qPCR, we found that Cflar_L_ mRNA exhibited no significant difference between HFD‐treated *Mef2c* ^*fl/fl*^ and *Emx1* ^*Cre*^ *Mef2c* ^*fl/fl*^ mice 3 days following ischemic stroke (Figure 4C), but Cflar_R_ was significantly diminished in HFD‐treated *Emx1* ^*Cre*^ *Mef2c* ^*fl/fl*^ mice compared to that in HFD‐treated *Mef2c* ^*fl/fl*^ mice 3 days following MCAO (Figure 4D). These results suggested Mef2c‐mediated alternative splicing of Cflar and promoted Cflar_R_ generation in the ischemic brain of HFD‐treated mice.

To examine Mef2c's impact on neuronal Cflar_R_ splicing at protein level, we co‐stained NeuN and cFLIP in the brain sections. We found that the percentage of cFLIP^+^ NeuN^+^ neuron was significantly descended in the peri‐infarct region of HFD‐treated *Emx1* ^*Cre*^ *Mef2c* ^*fl/fl*^ mice compared to that in HFD‐treated *Mef2c* ^*fl/fl*^ mice 3 days after MCAO (Figure 4E). To quantify the expression level of cFLIP isoforms in ischemic cortex, we found that the expression level of cFLIP_L_ did not significantly change between HFD‐treated *Emx1* ^*Cre*^ *Mef2c* ^*fl/fl*^ mice and *Mef2c* ^*fl/fl*^ mice, while cFLIP_R_ was significantly downregulated in HFD‐treated *Emx1* ^*Cre*^ *Mef2c* ^*fl/fl*^ mice compared to that in HFD‐treated *Mef2c* ^*fl/fl*^ mice 3 days following MCAO (Figure 4F). Moreover, we examined the expression level of cFLIP isoforms in the ischemic cortex of ND‐treated *Mef2c* ^*fl/fl*^ and *Emx1* ^*Cre*^ *Mef2c* ^*fl/fl*^ mice. We found that cFLIP_L_ did not show significant changes between ND‐treated *Emx1* ^*Cre*^ *Mef2c* ^*fl/fl*^ mice and *Mef2c* ^*fl/fl*^ mice 3 days following MCAO, while we detect no measurable cFLIP_R_ chemiluminescence signal in both groups, suggesting Mef2c only mediated Cflar_R_ splicing in the context of HFD but not in ND (Figure S2C). All the above results suggested Mef2c‐mediated neuronal Cflar alternative splicing and elevated cFLIP_R_ expressing level following ischemic stroke in the context of HFD.

### 3.5 Neuronal *Mef2c* Deficiency Improves Neuronal Survival and Functional Outcome in HFD Mice After Stroke

**FIGURE 5:**
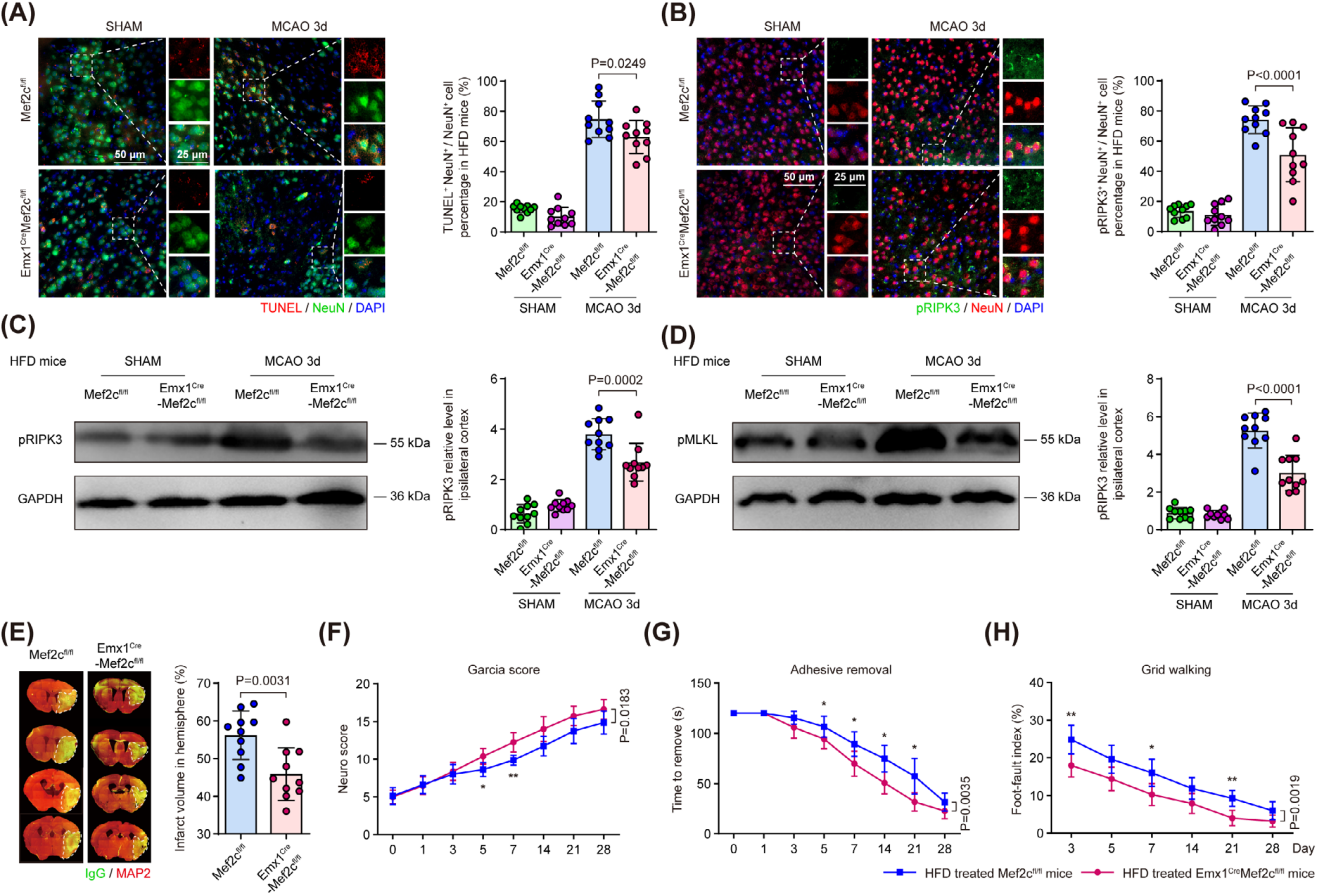
*Emx1* ^*Cre*^ *Mef2c* ^*fl/fl*^ exhibited mitigated neuronal necroptosis and improved long‐term neurological outcomes following MCAO in the context of HFD. (A, B) Representative images of neuron necroptosis in *Mef2c* ^*fl/fl*^ and *Emx1* ^*Cre*^ *Mef2c* ^*fl/fl*^ mice (both HFD‐treated) 3 days following MCAO. Quantification of TUNEL^+^ NeuN^+^ (A) and pRIPK3^+^ NeuN^+^ (B) neuron percentage in the peri‐infarct region. *n* = 10, One‐way ANOVA with Tukey's multiple comparison test. (C, D) Representative immunoblots for necroptotic kinases level of *Mef2c* ^*fl/fl*^ and *Emx1* ^*Cre*^ *Mef2c* ^*fl/fl*^ mice (both HFD‐treated) 3 days following MCAO. Quantitative analysis of pRIPK3 (C) and pMLKL (D) relative level in ischemic cortex. *n* = 10, One‐way ANOVA with Tukey's multiple comparison test. (E) Representative images and quantification of IgG and MAP2 double staining in the brain of *Mef2c* ^*fl/fl*^ and *Emx1* ^*Cre*^ *Mef2*c^*fl/fl*^ mice 3 days following MCAO (both HFD‐treated). *n* = 10, unpaired Student's *t‐*test. (F, H) Post‐stroke neurological assessment, including modified Garcia Score (F), adhesive removal test (G) and forelimb foot‐faults index (H), in *Mef2c* ^*fl/fl*^ and *Emx1* ^*Cre*^ *Mef2c* ^*fl/fl*^ mice (both HFD‐treated) through 28 days following MCAO. *n* = 8, Two‐way ANOVA with *post hoc* Bonferroni test, *p < 0.05, **p < 0.01 HFD MCAO versus ND MCAO. All data are presented as means ± SD.

To further verify that neuronal Cflar_R_ splicing isoform upregulated by Mef2c could aggravate post‐stroke necroptosis in the context of HFD, we employed immunostaining and western blot to measure the expression level of necroptotic markers. We found that the percentages of TUNEL^+^ NeuN^+^ and pRIPK3^+^ NeuN^+^ neuron in peri‐infarct regions, as well as the protein level of pRIPK3 and pMLKL in cerebral cortex were significantly decreased in HFD‐treated *Emx1* ^*Cre*^ *Mef2c* ^*fl/fl*^ mice compared to those in HFD‐treated *Mef2c* ^*fl/fl*^ mice 3 days following MCAO (Figure 5A–D). When the mice were treated with ND, we found that the percentage of TUNEL^+^ NeuN^+^ and pRIPK3^+^ NeuN^+^ neurons or the protein level of pRIPK3 and pMLKL in cerebral cortex were not significantly changed between *Mef2c* ^*fl/fl*^ and *Emx1* ^*Cre*^ *Mef2c* ^*fl/fl*^ mice 3 days following MCAO (Figure S3A–D). These results suggested that Mef2c was critical to neuronal necroptosis following ischemic stroke in the context of HFD but not ND.

To further evaluate the impact of neuronal specific Mef2c depletion on the ischemic brain injury and post‐stroke neurological functions, we subjected HFD‐treated *Mef2c* ^*fl/fl*^ or *Emx1* ^*Cre*^ *Mef2c* ^*fl/fl*^ mice to MCAO, and examined the brain injury with MAP2 and IgG double staining and behavioral tests. We found that the infarct volume was significantly reduced in HFD‐treated *Emx1* ^*Cre*^ *Mef2c* ^*fl/fl*^ mice compared to that in HFD‐treated *Mef2c* ^*fl/fl*^ mice 3 days following MCAO (Figure 5E). We found significant behavioral improvement in HFD‐treated *Emx1* ^*Cre*^ *Mef2c* ^*fl/fl*^ mice compared to *Mef2c* ^*fl/f*l^ control mice, consistently demonstrated in the mGarcia score, the adhesive removal test and the grid walking test (Figure 5F–H). These results suggest Mef2c depletion as a potential therapeutic candidate to improve neurological outcome after ischemic stroke with hyperlipidemia.

## 4 Discussion

In this study, we reported that hyperlipidemia exacerbated ischemic brain injury after stroke, and highlighted the novel functions of Mef2c in modulating alternative splicing of Cflar to further aggravate neuron necroptosis in HFD‐treated MCAO mice. Our findings reveal a novel mechanism of how hyperlipidemia comorbidity exacerbates ischemic brain injury after stroke and identify neuronal Cflar_R_ splicing regulated by Mef2c as a new therapeutic target for neuroprotection of stroke patients with hyperlipidemia.

Ischemic stroke with comorbid hyperlipidemia is emerging as a major global health concern [1]. Unveiling the mechanism that underlies the exacerbated ischemic brain injury of stroke with hyperlipidemia is of great importance to the development of novel treatment strategies for these patients. Using a HFD MCAO mouse model, we found that necroptosis is a crucial cell death pattern in ischemic neurons. The cascade process of necroptosis ends with MLKL phosphorylation and forming ion channels to interrupt cellular osmotic balance and membrane integration [10]. Inhibiting neuron necroptosis with Necrostatin‐1 or gene editing prevents reactive oxygen species and intracellular DAMPs from being released by neurons following ischemic stroke, which relieves secondary neuroinflammation and improved neurological recovery [11, 45]. Here, we demonstrated that increased neuron necroptosis is an important mechanism that underlies the HFD exacerbated ischemic brain injury in stroke with hyperlipidemia. It is of great importance to reveal how HFD exacerbate neuron necroptosis following ischemic stroke.

Mechanistically, we found that neuronal Cflar, an up‐stream regulator of necroptosis, was alternatively spliced in ischemic stroke with hyperlipidemia. HFD upregulated the level of pro‐necroptotic Cflar_R_ and cFLIP_R_ in ischemic neurons following MCAO. Accordingly, a previous study found that remote limb preconditioning downregulated the cFLIP_R_‐to‐cFLIP_L_ rate in salvage area, thus alleviating brain injury in rat model of permanent cerebral ischemia [22]. Another study revealed that RNA biding motif 5 coordinated alternative splicing process and suppressed cFLIP_S_ (structurally and functionally similar to cFLIP_R_) expression, leading to caspase activation in human neuronal SHSY5Y cells [37]. These findings suggested that Cflar splicing was a key step in determining neuronal cell death under acute stress. Moreover, hepatic cFLIP expression was upregulated in HFD‐treated mice, suggesting HFD also impacted on Cflar expression in other organs [46]. A cohort study included 374 pre‐school children from Europe for genome‐wide DNA‐methylation analysis, reporting that the methylation level of *CFLAR* was significantly correlated with fat mass and fat mass index [47]. Theses result suggest that fat intake and lipid metabolism could also regulate the epigenetic traits and expression level of *CFLAR* in stroke patients. AS Cflar_R_ triggers necroptosis, it is critical to figure out the modulation of post‐stroke Cflar_R_ splicing in the context of HFD.

Mef2c was predicted as a novel regulator of Cflar alternative splicing. We found that neuronal expression of Mef2c was upregulated by HFD 3 days following MCAO. Mef2c is categorized as a MADS box transcription factor that initiates multiple cell death processes and suppresses cell viability under stress [48, 49]. Mef2c ablation in retinal endothelial cells enhanced cellular resistance to hypoxia induced retinopathy, and Mef2c mediated the process of activation induced cell death in macrophages with the stimulation of lipopolysaccharide [50, 51]. Accordingly, we found that Mef2c silencing in ischemic neurons downregulated the levels of Cflar_R_, cFLIP_R_, pRIPK3, and pMLKL in HFD‐treated mice instead of ND‐treated mice 3 days following MCAO, which suggested that HFD aggravated neuron necroptosis in a Mef2c‐dependent manner. Moreover, the neurological outcome assessed by the mGarcia score, as well as the adhesive removal and grid walking tests were improved in HFD *Emx1* ^*Cre*^ *Mef2c* ^*fl/fl*^ mice up to 28 days following MCAO, suggesting ablation of neuronal Mef2c ameliorates post‐stroke neurological deficits especially. These findings demonstrated that Cflar splicing mediated by neuronal Mef2c was a critical pathogenic mechanism in hyperlipidemic stroke patients.

Notably, Mef2c has been reported to play multiple roles in neurons. A previous study reported neuronal Mef2c limited excessive synapse formation during synaptic refinement, while Mef2c was revealed to promote neuronal resilience against Alzheimer's disease (AD) pathology via preserving synapse integrity and plasticity [43, 52, 53]. Mef2c's function of maintaining synaptic plasticity and integrity could account for the approaching behavioral results of HFD‐treated *Emx1* ^*Cre*^ *Mef2c* ^*fl/fl*^ and *Mef2c* ^*fl/fl*^ in chronic phase of MCAO [54]. In addition, as lipid metabolism disorder and cerebral ischemic stroke were reported to alter epigenetic pattern in brain, the remodeling of Mef2c transcriptional network could explain Mef2c's mediating neuronal cell death and aggravating neurological deficits following ischemic stroke with concomitant hyperlipidemia [55, 56, 57]. These findings suggested that interventional strategies targeting Mef2c could help to precisely suppress the Mef2c‐associated pathogenic pathway if administered within a proper therapeutic time window. This may also enhance the impact of and outcome after recanalization therapy, so developing such strategies is in line with current recommendations [58].

There are several limitations of this study. First, the present study only focused on the Mef2c‐mediated alternative splicing of Cflar in ischemic neurons in the context of HFD, but the specific mechanism of how Mef2c interacted with spliceosome molecules and cis‐acting elements in *Cflar* gene sequence remains largely unknown. Further studies would investigate detailed mechanism of Cflar alternative splicing with immunoprecipitation, chromatin immunoprecipitation (ChIP), and X‐ray diffraction. Secondly, to demonstrate the alteration of Mef2c transcriptional network and other potential pathogenic mechanisms in HFD‐treated mice following MCAO, neuronal epigenetic landscape requires further elucidation. Multi‐omics analysis (including assay for transposase‐accessible chromatin using sequencing, methylated DNA immunoprecipitation, Mef2c related ChIP sequencing, and transcriptome) will be designed in our future studies. Thirdly, the present study only used male mice. Sexual difference exists in the pathophysiology of ischemic stroke, therefore, future studies on the role of Mef2c should also include female mice [59, 60].

## Author Contributions

T.H., R.L., and X.L. performed the experiments. R.L., J.Z., and X.L. performed the statistical analysis. R.L. and Y.G. performed bioinformatic analysis. Y.Z., F.L., and Y.L. performed experimental quality control for the current project. R.L. and P.L. wrote the manuscript. T.H. and Z.W. designed the experiments. J.W. supervised the project. P.L. and A.L. provided important comments to the project. All authors reviewed and approved the article.

## Conflicts of Interest

Peiying Li is an editorial board member of CNS Neuroscience and Therapeutics and a co‐author of this article. To minimize bias, they were excluded from all editorial decision‐making related to the acceptance of this article for publication.

## Supporting information


Data S1.

## Data Availability

The research data are available from the corresponding author on reasonable request.
