# Supplementary material for: Mef2c Exacerbates Neuron Necroptosis via Modulating Alternative Splicing of Cflar in Ischemic Stroke With Hyperlipidemia
Source: CNS Neurosci Ther. 2024 Dec 8;30(12):e70144. doi: 10.1111/cns.70144 (PMC11625962; doi:10.1111/cns.70144)
Supplement: Supplementary file 1 — Data S1. [file CNS-30-e70144-s001.docx]

**Mef2c exacerbates neuron necroptosis *via* modulating alternative splicing of Cflar in ischemic stroke with hyperlipidemia**

Ruqi Li^1^, Tianchen Huang^1^, Jianpo Zhou^1^, Xiansheng Liu^1^, Gan Li^1^, Yueman Zhang^2, 3^, Yunlu Guo^2, 3^, Fengshi Li^1^, Yan Li^2, 3^, Arthur Liesz^4, 5^, Peiying Li^2, 3,^, Zhenghong Wang^2^*^,^, Jieqing Wan^1^*

^1^Cerebrovascular Diseases Center, Department of Neurosurgery, Renji Hospital, Shanghai Jiao Tong University School of Medicine, Shanghai, China

^2^Department of Anesthesiology, Key Laboratory of the Ministry of Education, Renji Hospital, Shanghai Jiao Tong University School of Medicine, Shanghai, China

^3^Clinical Research Center, Renji Hospital, Shanghai Jiao Tong University School of Medicine, Shanghai, China

^4^Institute for Stroke and Dementia Research (ISD), University Hospital, LMU Munich, Germany

^5^Munich Cluster for Systems Neurology (SyNergy), Munich, Germany

R. Li and T. Huang are co-first authors and contributed equally to this work.

*Correspondence: Jieqing Wan, Cerebrovascular Diseases Center, Department of Neurosurgery, Renji Hospital, Shanghai Jiao Tong University School of Medicine, Shanghai 200127, China. Email: jieqingwan@126.com.

**Correspondence: Zhenghong Wang, Department of Anesthesiology, Renji Hospital, Shanghai Jiao Tong University School of Medicine, Shanghai 200127, China. Email: wangzhenghong@renji.com

**Supplemental Methods**

**Animals**

Wild-type C57BL/6 mice were purchased from GemPharmatech Co., China. To generate neuron cell-specific Mef2c knockout (*Emx1^Cre^Mef2c^fl/fl^*) mice, *Mef2c^fl/fl^* mice were crossed with *Emx1^Cre^* mice. Littermates (*Mef2c^fl/fl^*) were subjected to the control groups. The breeding and genotype examination of *Mef2c* transgenic mice were conducted by Cyagen Biosciences, China. Then, all mice were fed at the institute’s facility, in which maintains 22°C room temperature and 12-hour light/dark cycle. Sterilized food and water were free to access. A total of 355 mice were included in this study.

**Hyperlipidemia indecued by high fat diet**

To induce hyperlipidemia mode in mice, mice were randomly subjected to normal diet (ND) or high fat diet (HFD) treatment from 4 to 16 weeks old. HFD treated mice were provided with feeds containing high proportion of fat (60% energy supplied by fat), while ND treated mice were provided with feeds containing normal fat as the previous description.^1^ Body weights were measured weekly to evaluate the effects of HFD.

**Transient middle cerebral artery occlusion (MCAO)**

Cerebral ischemia was induced by endovascular occlusion of left middle cerebral artery (MCA). Mice were anesthetized with 2% isoflurane in a 30% O2 / 70% N2 mixture, while mice body temperature was maintained at 37°C ± 0.5°C with a heating pad throughout the surgery. A neck incision of 1 cm was made in the midline to expose left carotid artery (CA). Then, a monofilament covered with silicone was propelled through the external CA into the internal CA and up to the initial part of MCA, which led to blockage of MCA perfusion. The ischemia-inducing surgery was conducted by an experienced investigator, and the duration of surgery was strictly controlled within 10 to 15 minutes. The filament was pull out following the 60-minute ischemia to allow cerebral reperfusion. Mice in sham group underwent surgical procedures of artery exposure, but the MCA was not occluded. The duration of MCAO

Cerebral blood flow (CBF) was measured by a laser speckle imaging system (PeriCam PSI System, Perimed, Sweden) before and 10 minutes after ischemia. Mice were anesthetized and placed in a prone position during CBF measurement. Compared to baseline, animals exhibiting greater than 70% CBF reduction were considered to undergo successful MCAO procedures and included in this study.

**mRNA library construction**

Oligo(dT)-attached magnetic beads were used to purify mRNA. Purified mRNA was fragmented into small pieces with fragment buffer at an appropriate temperature. First-strand cDNA was generated using random hexamer-primed reverse transcription, followed by a second-strand cDNA synthesis. Afterwards, A-Tailing Mix and RNA Index Adapters were added to the incubation mix. cDNA fragments obtained from the previous step were amplified by PCR, and products were purified by Ampure XP Beads, then dissolved in EB solution. The product was validated on the Agilent Technologies 2100 bioanalyzer for quality control. The double stranded PCR products from the previous step were heat-denatured and circularized by a splint oligo sequence to get the final library. The single strand circle DNA (ssCir DNA) was formatted as the final library. The final library was amplified with phi29 to make DNA nanoball (DNB) which had more than 300 copies of one molecular, DNBs were loaded into the patterned nanoarray and pair end 100 bases reads were generated on BGIseq500 platform (Beijing Genomics Institute-Shenzhen, China).

**RNA sequencing analysis and transcription factor (TF) prediction**

The sequencing data was filtered with SOAPnuke (v1.5.2) to remove reads (i) containing sequencing adapter; (ii) whose low-quality base ratio (base quality less than or equal to 5) is more than 20%; (iii) whose unknown base (‘N' base) ratio is more than 5%. Thereafter, clean reads were obtained and stored in FASTQ format before being mapped to the reference genome using HISAT2 (v2.0.4). Then, Ericscript (v0.5.5) and rMATS (V3.2.5) were used to create fusion genes and differential splicing genes (DSGs) respectively. Bowtie2 (v2.2.5) was applied to align the clean reads to the gene set, a database for this brain tissue built by BGI (Beijing Genomic Institute in ShenZhen), which was known and novel, coding transcripts were included, then expression level of gene was calculated by RSEM (v1.2.12). Essentially, differential expression analysis was performed using the DESeq2(v1.4.5) with Q value ≤ 0.05 and |log_2_(Fold change)| ≥ 1. Volcano plot of differentially expressing genes (DEGs) was generated using ggpubr (v0.4.0) and ggplot2 (v3.4.2) through Hiplot Pro (https://hiplot.com.cn/). To gain insight into the change of phenotype, enrichment analysis of annotated DEGs and gene set enrichment analysis (GSEA) base on Kyoto Encyclopedia of Genes and Genomes (KEGG, https://www.genome.jp/kegg/) database were performed on Dr. Tom Multi-Omics Data Mining System (https://biosys.bgi.com/). Gene homology was calculated with SnapGene (v6.0.2). TF binding to *CFLAR* loci were predicted using TF-Target Finder (https://github.com/WangJin93/TFTF).^2^

**Immunofluorescence and BaseScope *in situ* hybridization**

Mice cerebral cryosections of sham and MCAO were selected to perform immunostaining. Sections were blocked with 10% donkey serum diluted by phosphate buffer saline with 1% Triton X-100 (1% PBST) at room temperature for 30 minutes, while sections were rinsed with 0.3% PBST. Primary antibodies were diluted to appropriate concentration by 0.3% PBST and incubated with the sections overnight at 4°C. Primary antibodies, including anti-Mef2c (1:300, Abcam Cat# ab211493, RRID:AB_2864417, UK), anti-cFLIP (1:300, Cell Signaling Technology Cat# 56343, RRID:AB_2799508, USA), anti-NeuN (1:500, Sigma-Aldrich Cat# MAB377, RRID:AB_2298772, German), anti-Iba-1(1:500, Abcam Cat# ab5076, RRID:AB_2224402), anti-GFAP (1:500, Cell Signaling Technology Cat# 3670, RRID:AB_561049), anti-MAP2 (1:500, Cell Signaling Technology Cat# 4542, RRID:AB_10693782) and anti-phosphorylated RIPK3 (1:300, Cell Signaling Technology Cat# 91702, RRID:AB_2937060) were utilized. Then, sections were incubation with appropriate fluorescent-labeled secondary antibodies (1:500, Abcam Cat# ab150065, RRID:AB_2860569; Abcam Cat# ab150105, RRID:AB_2732856; Abcam Cat# ab150076, RRID:AB_2782993; Abcam Cat# ab150112, RRID:AB_2813898; Abcam Cat# ab150136, RRID:AB_2782994) 1 hour at RT. Sections were covered with mounting medium containing antifading agents and DAPI (0100-20, SouthernBiotech, USA). All immunofluorescence images were captured by a laser scanning confocal system (Olympus Fluoview FV3000, Olympus, Japan). The number of target immunopositive cells and the radial immunofluorescence intensity were quantified using Image J (v1.52a, https://imagej.nih.gov/ij/). Three random fields were selected in the cortical peri-infarct area of each section for analysis by a blinded investigator.

BaseScope *in situ* hybridization of Cflar_L_ and Cflar_R_ splicing variants was performed on brain sections using BaseScope Duplex Reagent Kit (Advanced Cell Diagnostics, US) according to the manufacturer’s instruction.^3^ Probes for Cflar transcript variants were designed and provided by Advanced Cell Diagnostics. Sections were further stained with hematoxylin-eosin and observed in brightfield.

**TUNEL staining**

TdT-mediated dUTP nick end labeling (TUNEL) kit (G1502, Servicebio, China) was applied for TUNEL staining prior to the blocking step of co-labeling NeuN. In brief, cryosections were incubated with proteinase K (20 μg/ml) for 10 minutes at RT, and then washed with PBS 3 times. Reaction buffer was mixed by recombinant TdT enzyme, TMR-5-dUTP mix and equilibration buffer (1: 5: 50) and incubated with sections for 1 hour at RT, before the reaction was stopped by washing buffer for 4 times with 5 minutes each time.

**Infarct volume measurement**

Infarct volume was measured by Immunoglobulin G and Microtubule-associated protein 2 immunofluorescence with the procedures described above. Due to brain edema, the percentage of infarct volume was calculated by Σ [(contralateral hemisphere area - MAP2 positive area) × section interval] / Σ (contralateral hemisphere area × section interval) × 100%.

**Neurological function assessments**

Behavioral tests, including modified Garcia (mGarcia) score, grid walk test and adhesive removal test, were performed on 0 (12 hours), 1, 3, 5, 7, 14, 21, 28 days after MCAO. Animals’ identity and treatment were blinded the investigator who conducted neurological assessments. Detailed methods of behavioral assessments were listed as follow.

**Modified Garcia (mGarcia) Score:** 6 subitems of mGarcia score ranged from 0 to 3 or 1 to 3 (total points ranged from 3 to18) were measured: (a) body proprioception, (b) climbing, (c) forelimb walking, (d) limb symmetry, (e) response to vibrissae touch, and (f) spontaneous activity as described.^4^ Body proprioception and response to vibrissae touch reflected somatosensory function, while the rest of tests represented motor functions.The detailed scoring criteria described by Yeh et al. were employed to assess sensorimotor deficits in mice.^5^

**Grid walk test:** Mice were placed on a steel grid platform (30 cm × 40 cm), which was lifted 30 cm from the ground, freely to explore. Missing steps in 5 minutes were monitored to calculated foot-fault index by (right impaired forelimb faults - left intact forelimb faults) / total forelimb faults of bilateral forelimbs.^6^

**Adhesive removal test:** Experiment procedures were designed based on previous descriptions.^7^ In short, each adhesive sticker was cut into 0.3 × 0.4 cm^2^ and pressed on each forepaw of a mouse with same pressure. The order of sticking adhesive to left or right forepaw was randomized within each group. The mouse was then placed into a transparent cage. The average time needed to remove both tapes in each mouse was recorded. Mice were trained twice in 6 and 3 days prior to MCAO surgery.

**Quantitative PCR assay**

Cerebral cortex was carefully separated under stereo microscope following mice scarification. Total RNA was extracted from peri-infarct tissue by TRIzol reagent (R401-01, Vazyme, China) following closely to the manufacturer’s protocol. The HiScript II cDNA synthesis kit (R212-01, Vazyme) was employed for reverse transcription. The cDNA products were mixed with SYBR qPCR mix (Q711-02, Vazyme) and primers (Mef2c Forward 5’-3’: TCCTGGTGTAACACATAGACCTC; Reverse 5’-3’: TGGTAAAGTAGGAGTTGCTACGG; Cflar_L_ Forward 5’-3’: TCCGCACATCCGTGAAGAGACT; Reverse 5’-3’: ATGATAGCCCAGGGAAGTGAAGGT; Cflar_R_ Forward 5’-3’: GCCTGAAGAACATCCACAGAATAGAC; Reverse 5’-3’: TCATGCTGGTACTCCATACACTGG) for quantitative PCR (qPCR). Reaction conditions were as follows: pre-denaturation at 95°C for 30 seconds, 40 cycles of 95°C for 10 seconds following 60°C for 30 seconds, acquisition of melting curve, while reactions were performed on LightCycler 96 Instrument (Roche, Switzerland). Relative mRNA levels were quantified by normalization to the expression of the β-actin (Forward 5’-3’: GGCTGTATTCCCCTCCATCG; Reverse 5’-3’: CCAGTTGGTAACAATGCCATGT) and shown as fold changes to the control groups. Results were analyzed by a blinded investigator.

**Western blot**

Prepared ipsilateral lesion cortex of ischemia from sham and MCAO mice was placed in radioimmuno precipitation assay buffer (RIPA) solution (PC102, EpiZyme, China) containing a protease inhibitor cocktail (P1005, Beyotime, China) for lysis. Proteins samples were loaded on 10% polyacrylamide gels (20325ES62, Yeasen Biotech., China) and transferred onto a PVDF membrane (IPVH00010; Millipore) for 2 hours at 200 mA (constant). Following blocking with 5% milk for 1 hour at RT, the membrane was incubated with anti-cFLIP (1:500), anti-Mef2c (1:500), anti-phosphorylated RIPK3 (1:500), anti-phosphorylated MLKL (1:500, Cell Signaling Technology Cat# 37333, RRID:AB_2799112) and anti-GAPDH (1:10000, ABclonal Cat# AC035, RRID:AB_2769863, China). After overnight incubation of primary antibody at 4°C, membranes were incubated with secondary HRP-conjugated antibody (1:10000, Cell Signaling Technology Cat# 5127, RRID:AB_10892860) for 1 hour at RT. Chemiluminescence was performed on ChemiDoc Imaging System (Bio-Rad, US) using Immobilon Western HRP Substrate (Millipore). The optical density of the signals of stripes were quantified using grayscale measurement in Image J (v1.52a) and calculated to relative expression for analysis by a blinded investigator.

**Statistical analysis**

All statistics were performed on GraphPad Prism (v8, https://www.graphpad-prism.cn/) or implemented statistical tests in R packages. All results were continuous variables and presented as mean ± standard deviation (SD). The Shapiro-Wilk normality test was initially performed on all datasets. The unpaired Student’s *t* test was used for the comparison between two groups. The one-way analysis of variance (ANOVA) with Turkey’s multiple comparisons test was employed to examine variation among groups (more than 2 groups). Since the time following MCAO served as a covariate to neurological functions recovery, the two-way ANOVA with *post hoc* Bonferroni test was utilized for behavioral tests analysis. The threshold of *P* value was set at 0.05.

**REFERENCES**

1. Wang L, Zheng W, Yang J, Ali A, Qin H. Mechanism of Astragalus membranaceus Alleviating Acquired Hyperlipidemia Induced by High-Fat Diet through Regulating Lipid Metabolism. *Nutrients*. 2022;14(5).

2. Wang J. TFTF: An R-Based Integrative Tool for Decoding Human Transcription Factor-Target Interactions. *Biomolecules*. 2024;14(7).

3. Wang F, Flanagan J, Su N, Wang LC, Bui S, Nielson A, et al. RNAscope: a novel in situ RNA analysis platform for formalin-fixed, paraffin-embedded tissues. *J Mol Diagn*. 2012;14(1):22-9.

4. Garcia JH, Wagner S, Liu KF, Hu XJ. Neurological deficit and extent of neuronal necrosis attributable to middle cerebral artery occlusion in rats. Statistical validation. *Stroke*. 1995;26(4):627-34; discussion 35.

5. Yeh SJ, Tang SC, Tsai LK, Jeng JS, Chen CL, Hsieh ST. Neuroanatomy- and Pathology-Based Functional Examinations of Experimental Stroke in Rats: Development and Validation of a New Behavioral Scoring System. *Front Behav Neurosci*. 2018;12:316.

6. Rogers DC, Campbell CA, Stretton JL, Mackay KB. Correlation between motor impairment and infarct volume after permanent and transient middle cerebral artery occlusion in the rat. *Stroke*. 1997;28(10):2060-5; discussion 6.

7. Bouet V, Boulouard M, Toutain J, Divoux D, Bernaudin M, Schumann-Bard P, et al. The adhesive removal test: a sensitive method to assess sensorimotor deficits in mice. *Nat Protoc*. 2009;4(10):1560-4.

**Supplemental Tables**

**Table S1. Evaluation of transcription factors binding to *CFLAR* gene with JASPAR database.**

| TF | Target gene | Score | P.Value | Q.Value | Matched sequence |
| --- | --- | --- | --- | --- | --- |
| CTCF | *CFLAR* | 11.5596 | 9.91E-06 | 0.15 | GGGCAAGGATCACTTGAGGCCAGCAGTTGGAGA |
| CTCF | *CFLAR* | 10.6066 | 3.55E-05 | 0.26 | GCCAGCAGTTGGAGA |
| E2F6 | *CFLAR* | 11.2114 | 7.04E-05 | 0.27 | AGCGGGAA |
| E2F6 | *CFLAR* | 10.9268 | 7.86E-05 | 0.27 | GGCGGGCA |
| FOSL2 | *CFLAR* | 11.0364 | 5.81E-05 | 0.37 | GGTGGCTCAC |
| FOSL2 | *CFLAR* | 11.0364 | 5.81E-05 | 0.37 | GGTGGCTCAC |
| FOXA1 | *CFLAR* | 11.028 | 7.93E-05 | 1 | GTAAACAC |
| FOXA1 | *CFLAR* | 11.028 | 7.93E-05 | 1 | GTAAACAC |
| FOXA2 | *CFLAR* | 13.2254 | 2.18E-05 | 0.87 | TGTAAACA |
| FOXA2 | *CFLAR* | 12.9014 | 4.35E-05 | 0.88 | AGTAAACA |
| GABPA | *CFLAR* | 10.4929 | 9.04E-05 | 0.39 | CCTTTCCTGT |
| IRF4 | *CFLAR* | 11.1364 | 3.40E-06 | 1 | TGAAACTGAAAATT |
| MAFK | *CFLAR* | 13.2061 | 1.40E-05 | 0.11 | CTGCCTCAGC |
| MAFK | *CFLAR* | 10.5212 | 7.95E-05 | 0.34 | GTGTCTCAGC |
| MAFK | *CFLAR* | 10.1455 | 9.63E-05 | 0.37 | TTGCCTCAGC |
| **MEF2C** | ***CFLAR*** | **13.1017** | **1.50E-05** | **0.13** | **CTAAAAATACA** |
| **MEF2C** | ***CFLAR*** | **13.1017** | **1.50E-05** | **0.13** | **CTAAAAATACA** |
| PAX5 | *CFLAR* | 11.4455 | 5.98E-05 | 1 | GCGAGACC |
| RELA | *CFLAR* | 13.3061 | 2.01E-05 | 0.56 | GGGAGATTCC |
| RELA | *CFLAR* | 11.1735 | 4.86E-05 | 0.61 | CTGGATTTCC |
| RUNX3 | *CFLAR* | 12.0567 | 5.04E-05 | 0.68 | AACCACAA |
| SP1 | *CFLAR* | 14.3776 | 1.07E-05 | 0.04 | GGGGCGCGG |

Each line showed one independent record of predicted TF binding site in *CFLAR* gene. Records of MEF2C were highlighted in bold. Transcription factor (TF); CASP8 and FADD-like apoptosis regulator (CFLAR); CCCTC-binding factor (CTCF); E2F transcription factor 6 (E2F6); FOS like 2, AP-1 transcription factor subunit (FOSL2); Forkhead box A1 (FOXA1); Forkhead box A2 (FOXA2); GA binding protein transcription factor subunit alpha (GABPA); Interferon regulatory factor 4 (IRF4); MAF bZIP transcription factor K (MAFK); Myocyte enhancer factor 2 (MEF2C); Paired box 5 (PAX5); RELA proto-oncogene, NF-kB subunit (RELA); RUNX family transcription factor 3 (RUNX3); Sp1 transcription factor (SP1).

**Supplemental Figures**

**Figure S1. The expression of Mef2c was not significantly changed in astrocytes or microglia in HFD treated mice.**


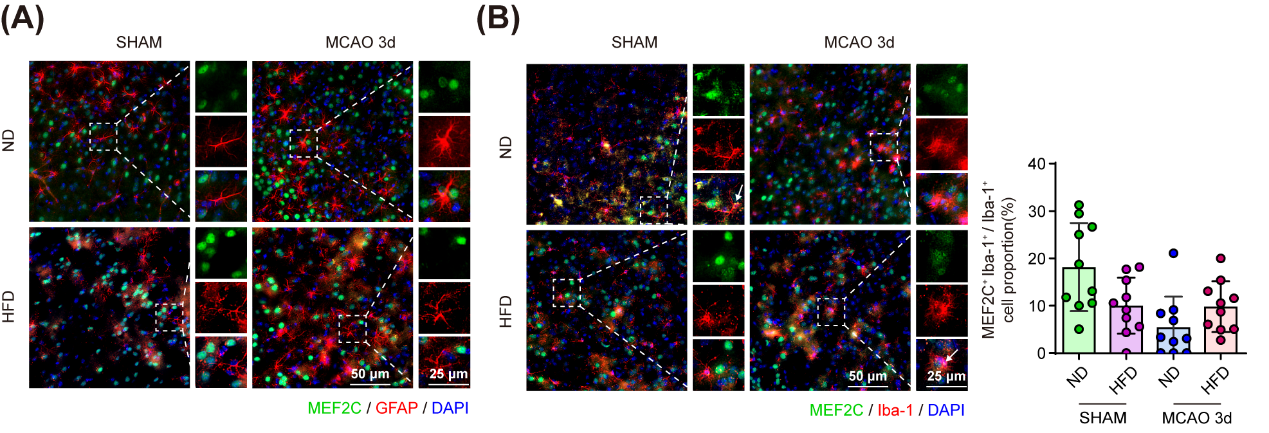


**Figure S1. The expression of Mef2c was not significantly changed in astrocytes or microglia in HFD treated mice.**

(A) Representative image demonstrated Mef2c expression in astrocyte of ND and HFD mice in 3 days after MCAO. (B) Representative image of microglial Mef2c expression in ND and HFD mice on the third day after MCAO. Quantitative measurement of Mef2c^+^ Iba-1^+^ microglia percentage. n = 10, one-way ANOVA with Tukey’s multiple comparison test. All data were presented as means ± SD. High-fat diet (HFD), normal diet (ND); middle cerebral artery occlusion (MCAO); Analysis of Variance (ANOVA); Standard deviation (SD).

**Figure S2.** ***Emx1^cre^Mef2c^fl/fl^* mice with normal diet did not significantly change cerebral cFLIP expression following MCAO.**


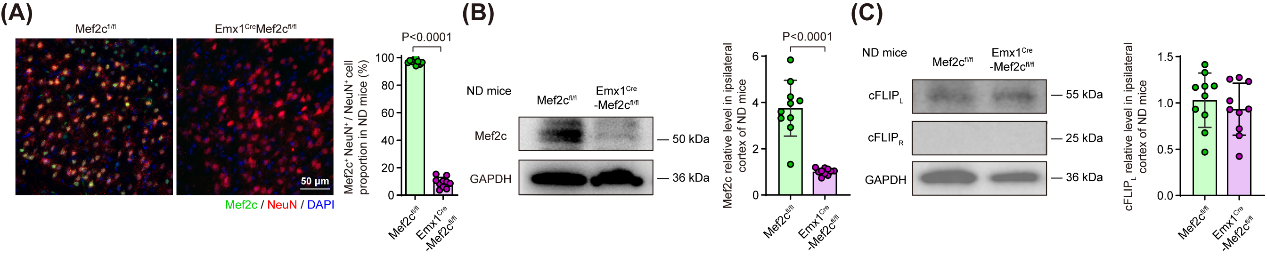


**Figure S2.** ***Emx1^cre^Mef2c^fl/fl^* mice with normal diet did not significantly change cerebral cFLIP expression following MCAO.**

(A) Representative images showing Mef2c expression in neurons of *Mef2c^fl/fl^* and *Emx1^Cre^Mef2c^fl/fl^* mice fed with normal diet (ND) 3 days following MCAO. Quantitative analysis of Mef2c^+^ NeuN^+^ neuron percentage in the peri-infarct region. n = 10, unpaired Student’s *t* test. (B) Representative immunoblot for cortical Mef2c level of *Mef2c^fl/fl^* and *Emx1^Cre^Mef2c^fl/fl^* mice (both ND treated) 3 days following MCAO. Quantitative analysis of Mef2c relative level in ischemic cortex. n = 10, unpaired Student’s *t* test. (C) Representative immunoblot for cortical cFLIP_L_ and cFLIP_R_ level of *Mef2c^fl/fl^* and *Emx1^Cre^Mef2c^fl/fl^* mice (both ND treated) 3 days following MCAO. Quantitative analysis of cFLIP_L_ relative level in ischemic cortex. n = 10, unpaired Student’s *t* test. All data were presented as means ± SD.

**Figure S3.** ***Emx1^cre^Mef2c^fl/fl^* mice with normal diet did not exhibit significant changes in neuron necroptosis and neurological deficits following MCAO.**


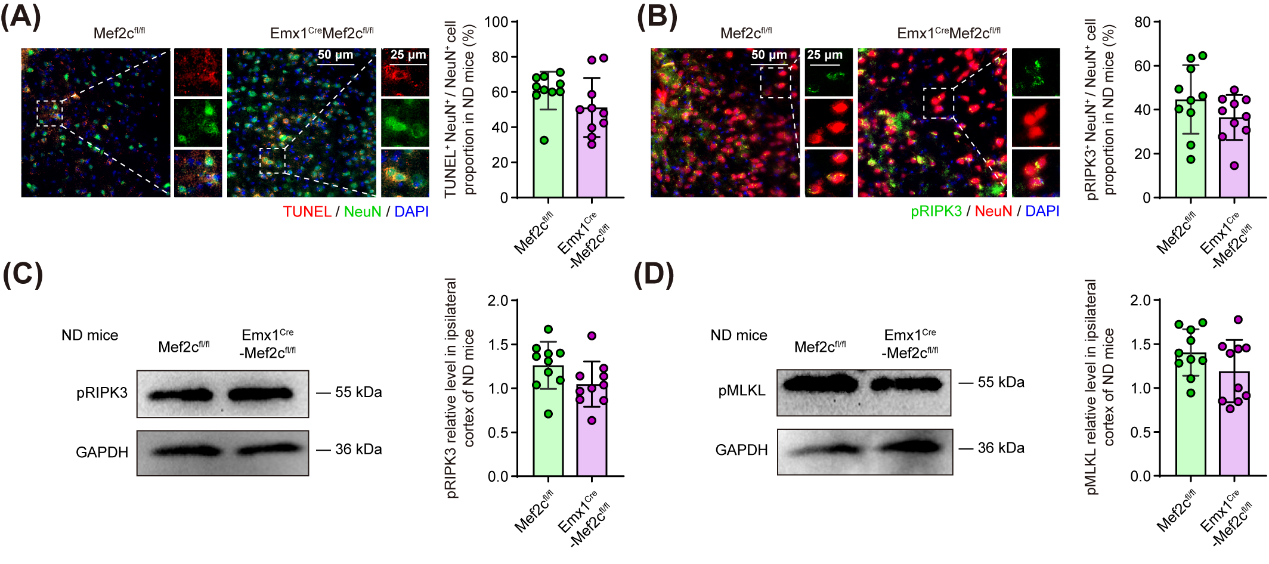


**Figure S3.** ***Emx1^cre^Mef2c^fl/fl^* mice with normal diet did not exhibit significant changes in neuron necroptosis and neurological deficits following MCAO.**

(A-B) Representative images of neuron necroptosis in *Mef2c^fl/fl^* and *Emx1^Cre^Mef2c^fl/fl^* mice (both ND treated) 3 days following MCAO. Quantification of TUNEL^+^ NeuN^+^ (A) and pRIPK3^+^ NeuN^+^ (B) neuron percentage in the peri-infarct region. n = 10, unpaired Student’s *t* test. (C-D) Representative immunoblots for necroptotic kinases level of *Mef2c^fl/fl^* and *Emx1^Cre^Mef2c^fl/fl^* mice (both ND treated) 3 days following MCAO. Quantitative analysis of pRIPK3 (C) and pMLKL (D) relative level in ischemic cortex. n = 10, unpaired Student’s *t* test. All data were presented as means ± SD.
